# Identification of superior late‐blooming apricot (*Prunus armeniaca* L.) genotypes among seedling‐originated trees

**DOI:** 10.1002/fsn3.2747

**Published:** 2022-01-18

**Authors:** Zeinab Mashhadi, Ali Khadivi

**Affiliations:** ^1^ Department of Horticultural Sciences, Faculty of Agriculture and Natural Resources Arak University Arak Iran

**Keywords:** apricot, breeding, cultivation, fruit quality, late blooming

## Abstract

One of the major limiting factors in the intensive spread of apricot (*Prunus armeniaca* L.) in most of the countries is spring frost. Thus, the breeding efforts have concentrated on the use of late‐blooming genotypes as a means of frost avoidance. The aim of the present study was to identify late‐blooming genotypes with high fruit quality among seedling‐originated trees. Firstly, pre‐selections were done according to blooming time within 278 apricot seedling‐originated trees. Secondly, the late‐blooming selections were further evaluated according to their vegetative and fruit characteristics to determine superior types. Significant differences were observed among the late‐blooming genotypes in terms of the traits recorded. Fruit ground color was strongly variable, including white, yellow, yellow–green, light orange, orange, and dark orange. Fruit weight ranged from 27.37 to 33.99 g, fruit flesh thickness varied from 11.16 to 13.47 mm, and total soluble solids varied between 17.00% and 23.87%. Hierarchical cluster analysis (HCA) performed with Euclidean distance and Ward's method divided the genotypes into two main clusters based on morphological traits and in some cases, the genotypes belonging to an area were clustered into different clusters. All the 48 late‐blooming date genotypes selected could be useful as a parent to improve flowering season of cultivars. Also, among them, 10 genotypes were superior in terms of fruit quality‐related characters, such as fruit weight, fruit color, fruit taste, and TSS, and thus they can be singled out for cultivation.

## Graphical abstract

All the 48 late‐blooming date apricot (*Prunus armeniaca* L.) genotypes could be useful as a parent to improve flowering season of cultivars. Also, among them, 10 genotypes were superior in terms of fruit quality‐related characters, such as fruit weight, fruit color, and fruit taste, and thus they can be singled out for cultivation.

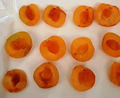

## 1 INTRODUCTION

Apricot (*Prunus armeniaca* L.) is one of the most popular temperate fruit trees grown over the world (Faust et al., 1998). It belongs to the family Rosaceae, subfamily Pronoideae, genus *Prunus*, subgenus *Prunuphora* Focke, and section Armeniaca (Lam.) Koch. The generic term apricot includes four different species, viz, *P. armeniaca* L., the cultivated apricot; *P. sibirica* L., the Siberian apricot; *P. mandshurica* (Maxim.) Koehne, the Manchurian apricot; *P. mume* (Siebold) Siebold & Zucc., the Japanese apricot, and one naturally occurring interspecific hybrid, *Prunus* ×dasycarpa Ehrh., the black or purple apricot (Rehder, 1949). Among these, *P. armeniaca* is the most widely cultivated (Uzun et al., 2010), with highest global production.

Apricot cultivars are divided into four eco‐geographical groups: Central Asian, Irano‐Caucasian, European, and Dzhungar‐Zailing (Lopes et al., 2002). The Central Asian group is the oldest group with the richest variation. Most of the cultivars are self‐incompatible with small‐to‐medium‐sized fruits which ripen over a long period and require high chilling (Uzun et al., 2010).

Apricot fruits are a great source of many antioxidants, including beta‐carotene and vitamins A, C, and E. The main flavonoids in apricots are chlorogenic acids, catechins, and quercetin (Haciseferogullari et al., 2007). Apricot kernels are used as roasted and salted titbit. The kernel is a rich source of dietary protein, oil, and fiber. Kernels are a good source of fatty acids and phenolic compounds. The kernels are considered as nontraditional potential resources for oils. Large quantities of fruit kernels are usually discarded by the food processing industry (Özcan, 2000; Matthaus & Özcan, 2009; Matthaus et al., 2016; AL‐Juhaimi et al., 2018).

The development of new fruit cultivars generally has been based on genetic resources. Germplasm collection and characterization are essential stages of breeding programs. Given the importance of apricot industry for this unique ecogeographic zone, characterization of germplasm collections and genetic diversity analysis are a prerequisite for any breeding program. Genetic resource management, including collection, precise characterization, and documentation of extant variability is of paramount importance for conservation, breeding, and commercialization of potential apricot genotypes. Morphological characterization is timely for cultivar identification, selection, delineation, and germplasm management in start‐up programs intended for selection of superior genotypes for breeding programs. Several studies have been undertaken on the variability of germplasm resources of European (Audergon et al., 1991; Badenes et al., 1998; Milosevic et al., 2010) and Irano‐Caucasian eco‐geographical groups (Asma et al., 2007; Asma & Ozturk, 2005), resulting in the identification of interesting cultivars that have been used to generate new selections through breeding programs (Almeras et al., 2002; Forte, 1971; Krichen et al., 2014a,2014b).

One of the major limiting factors in the intensive spread of apricot in most of the countries, including Iran, is spring frost which kills blossoms. Apricot cultivars with late flowering can be cultivated in mountain areas, where the late frosts are frequent. The breeding efforts have concentrated on the use of delayed flowering as a means of frost avoidance. The number of studies on Iranian apricot germplasm is limited (Khadivi‐Khub & Khalili, 2017; Rezaei et al., 2020). The aim of the present study was to identify late‐blooming apricot genotypes with high fruit quality among seedling‐originated trees in the Markazi province/Iran.

## 2 MATERIALS AND METHODS

### 2.1 Plant material

The present study was undertaken to assess the genetic diversity in seedling‐originated apricot trees grown through morphological characters and to identify late‐blooming genotypes with high fruit quality in the Markazi province/Iran. Firstly, pre‐selections were done according to blooming time within 278 apricot seedling‐originated trees from three areas which were near each other. The genotypes with early and middle blooming dates were eliminated and finally, 48 late‐blooming trees were selected. Secondly, the late‐blooming selections were further evaluated according to their vegetative and fruit characteristics to determine superior types. The selected genotypes were named based on their location, and these names were supplemented with numerical characters. The selected trees were mature (8–10 years old), healthy, and had a full crop. General orchard management, including irrigation, nutrition, pest, and disease control, was consistent with commercial practices.

### 2.2 The characters evaluated

The selected late genotypes were evaluated using 47 morphological and pomological traits to select superior selections. Length, width, and thickness for leaf, fruit, stone, and kernel were measured using a digital caliper. Weight for fruit, stone, and kernel was measured using an electronic balance with 0.01 g precision. Total soluble solids (TSS) content was determined using a refractometer (pocket PAL‐1 ATAGO Corporation, Tokyo, Japan), in ◦Brix. The remaining characters were qualitatively determined based on rating and coding according to the apricot descriptor (Guerriero & Watkins, 1984, IBPGR).

### 2.3 Statistical analysis

Analysis of variance (ANOVA) was performed to evaluate the variation among the genotypes based on the traits measured using SAS software (SAS Institute, Cary, NC, USA, 1990). Simple correlations between traits were determined using Pearson correlation coefficients (SPSS Inc., Chicago, IL, USA, Norusis, 1998). Principal component analysis (PCA) was used to investigate the relationship between genotypes and determine the main traits effective in genotype segregation using SPSS software. Hierarchical cluster analysis (HCA) was performed using Ward's method and Euclidean coefficient using PAST software (Hammer et al., 2001). The first and second principal components (PC1/PC2) were used to create a scatter plot with PAST software.

## 3 RESULTS AND DISCUSSION

Firstly, pre‐selections were done according to blooming time within 278 apricot seedling‐originated trees. The blooming time of the 278 trees studied ranged from 10 March to 09 April. Thus, the blooming time of 278 trees was as very early (20 genotypes), early (62 genotypes), moderate (148), and late (48). Significant variabilities in blooming time of genotypes under the same geographical conditions might be a result of the total exposure temperature required. Full blooming immediately follows the end of the dormancy period (Blasse & Hofmann, 1993). Temperatures ranging from 7 to 9˚C determine the start of the phenophase “beginning of blossoming” (Vachun, 2003). The difference in the flowering time between the genotypes is 2–4 days under favorable environmental conditions or 6–8 days under less favorable ones (Milosevic, 1997). Late blooming is an important factor to protect damages caused by spring frosts in continental climates (Unal et al., 1999). Therefore, finding late‐blooming trees is one of the main goals of apricot breeding program. Thus, secondly, the 48 late‐blooming genotypes selected were evaluated according to their vegetative and fruit characteristics to determine superior types.

There were significant differences among the late‐blooming genotypes selected in terms of the traits recorded. The CV ranged from 1.59 (spur leaf width) to 90.18% (ripening date). The CV was more than 20.00% in 28 out of 47 characters recorded (Table 1).

**TABLE 1:** Statistical descriptive parameters for morphological traits used to study late‐blooming apricot genotypes

No.	Character	Unit	Min.	Max.	Mean	*SD*	CV (%)
1	Tree growth habit	Code	1	3	1.62	0.94	57.84
2	Tree growth vigor	Code	3	5	3.71	0.97	26.06
3	Tree height	Code	3	5	3.79	0.99	26.07
4	Trunk color	Code	1	3	1.83	1.00	54.43
5	Trunk diameter	Code	3	5	4.04	1.01	25.00
6	Canopy density	Code	3	5	4.04	1.01	25.00
7	Branching	Code	3	5	4.00	1.01	25.28
8	Branch density	Code	3	5	3.96	1.01	25.51
9	Branch flexibility	Code	1	3	1.54	0.90	58.31
10	Branch leaf length	mm	48.24	57.88	52.44	2.13	4.07
11	Branch leaf width	mm	51.85	67.12	59.74	3.43	5.74
12	Branch petiole length	mm	24.61	31.64	26.07	1.04	3.99
13	Spur leaf length	mm	73.29	79.14	75.58	1.21	1.60
14	Spur leaf width	mm	71.36	77.28	74.74	1.19	1.59
15	Spur petiole length	mm	17.72	19.51	18.73	0.44	2.34
16	Leaf density	Code	1	5	2.79	1.18	42.44
17	Leaf shape	Code	1	3	1.50	0.88	58.33
18	Leaf serration shape	Code	1	3	1.62	0.94	57.84
19	Leaf upper surface color	Code	1	5	3.38	1.41	41.69
20	Leaf lower surface color	Code	1	3	1.54	0.90	58.31
21	Leaf apex shape	Code	1	3	2.00	1.01	50.55
22	Fruit yield	Code	1	5	1.92	1.09	56.67
23	Ripening date	Date	Late May	Early June	1.67	1.51	90.18
24	Fruit apex shape	Code	1	3	2.42	0.92	37.98
25	Fruit shape	Code	1	5	3.88	1.75	45.00
26	Fruit length	mm	34.99	38.20	36.32	0.80	2.19
27	Fruit width	mm	31.32	36.88	34.11	1.46	4.27
28	Fruit weight	g	27.37	33.99	30.38	1.79	5.89
29	Fruit flesh thickness	mm	11.16	13.47	12.35	0.68	5.49
30	Fruit ground color	Code	1	11	4.58	3.11	67.99
31	Fruit flesh color	Code	1	9	3.33	1.95	58.56
32	Fruit flesh softness	Code	1	7	3.29	1.54	46.90
33	Stone length	mm	28.13	30.92	29.14	0.67	2.31
34	Stone width	mm	20.65	24.37	22.39	0.80	3.57
35	Stone thickness	mm	10.40	11.78	11.02	0.33	2.95
36	Stone weight	g	1.48	2.71	1.95	0.23	11.64
37	Flesh adhesion to stone	Code	1	5	2.62	1.58	60.27
38	Total soluble solids	%	17.00	23.87	20.95	1.38	6.59
39	Kernel length	mm	18.27	20.19	19.12	0.53	2.75
40	Kernel width	mm	13.62	17.51	15.54	0.86	5.55
41	Kernel thickness	mm	6.65	7.41	7.02	0.16	2.31
42	Kernel weight	g	0.70	0.81	0.75	0.03	3.49
43	Kernel shape	Code	1	3	2.62	0.79	30.11
44	Kernel color	Code	1	7	3.17	1.64	51.80
45	Kernel shriveling	Code	1	5	2.25	1.41	62.53
46	Kernel pubescence	Code	1	3	1.21	0.62	50.99
47	Kernel taste	Code	1	5	2.58	1.37	52.95

Tree growth habit was standard (33 genotypes) and open (15). Tree growth vigor, tree height, branch density, and leaf density were predominantly moderate (Table 2). The range of leaf‐related dimensions was as follows: branch leaf length: 48.24–57.88 mm, branch leaf width: 51.85–67.12 mm, branch petiole length: 24.61–31.64 mm, spur leaf length: 73.29–79.14 mm, spur leaf width: 71.36–77.28‐mm, and spur petiole length: 17.72–19.51 mm (Table 1). The tree size and vegetative growth are affected by genetic and ecological factors (Asma & Ozturk, 2005).

**TABLE 2:** Frequency distribution for the measured qualitative morphological characters in the studied late‐blooming apricot genotypes

Character	Frequency (no. of genotypes)					
	1	3	5	7	9	11
Tree growth habit	Standard (33)	Open (15)	–	–	–	–
Tree growth vigor	–	Moderate (31)	High (17)	–	–	–
Tree height	–	Moderate (29)	High (19)	–	–	–
Trunk color	Red (28)	Black (20)	–	–	–	–
Trunk diameter	–	Moderate (23)	High (25)	–	–	–
Canopy density	–	Moderate (23)	High (25)	–	–	–
Branching	–	Moderate (24)	High (24)	–	–	–
Branch density	–	Moderate (25)	High (23)	–	–	–
Branch flexibility	Low (35)	Moderate (13)	–	–	–	–
Leaf density	Low (11)	Moderate (31)	High (6)	–	–	–
Leaf shape	Flat (36)	Oblong (12)	–	–	–	–
Leaf serration shape	Tiny (33)	Moderate (15)	–	–	–	–
Leaf upper surface color	Light green (8)	green (23)	Dark green (17)	–	–	–
Leaf lower surface color	Light green (35)	green (13)	–	–	–	–
Leaf apex shape	Blate (24)	Acute (24)	–	–	–	–
Fruit yield	Low (27)	Moderate (20)	High (1)	–	–	–
Ripening date	Late May (40)	Early June (8)	–	–	–	–
Fruit apex shape	Blate (14)	Acute (34)	–	–	–	–
Fruit shape	Flat (12)	Oval (3)	Oblong (33)	–	–	–
Fruit ground color	White (11)	Yellow (15)	Yellow–green (7)	Light orange (6)	Orange (6)	Dark orange (3)
Fruit flesh color	White (8)	Yellow (32)	Yellow–green (3)	Light orange (2)	Orange (3)	
Fruit flesh softness	Soft (9)	Moderate (25)	Firm (12)	Very Firm (2)	–	–
Flesh adhesion to stone	Low (20)	Moderate (17)	High (11)	–	–	–
Kernel shape	Flat (9)	Oblong (39)	–	–	–	–
Kernel color	Very light (12)	Light (22)	Moderate (12)	Dark (2)	–	–
Kernel shriveling	Low (24)	Moderate (18)	High (6)	–	–	–
Kernel pubescence	Low (43)	Moderate (5)	–	–	–	–
Kernel taste	Bitter (17)	Moderate (24)	Sweet (7)	–	–	–

Ripening date ranged from late May (40 genotypes) to early June (8). Extending the ripening season is interesting in breeding programs of apricot. The influence of growing degree‐day thresholds on ripening time is very important for apricot‐producing regions (Ruml et al., 2010). Fruit shape was predominantly oblong (33 genotypes). Fruit shape and size determine market value and are important physical attributes in grading, sorting, packaging, and transportation of fruits (Erdogan et al., 2003).

Fruit ground color was strongly variable, including white (11), yellow (15), yellow–green (7), light orange (6), orange (6), and dark orange (3). Also, there was significant diversity among the genotypes in terms of fruit flesh color, ranging from white to orange (Table 2). The fruit color is an important indicator of fruit ripeness and harvest date of some fruits. Also, the cultivars with different fruit peel colors can be satisfying various consumer preferences (Caliskan & Polat, 2011). Two main factors, including genotype and stage maturity, are influencing the evolution of fruit color parameters (Ayour et al., 2016). Ruiz et al. (2005) reported that carotenoid content in apricot fruit showed significant correlations with skin and flesh color, with apricots having orange‐colored flesh containing higher levels of carotenoids than those having white‐colored flesh. It has been shown that the orange color was closely correlated with the carotenoid content (Marty et al., 2005). Previous studies focused on chlorophylls degradation and showed that this degradation was accompanied by the formation of chromo‐plastids during fruit ripening (Abaci & Asma, 2013). Chlorophyll degradation during maturation occurs in parallel with the development and accumulation of other pigments, such as carotenoids. It has been reported that beta‐carotene is the main pigment quantified in apricot fruit (Ayour et al., 2016; Munzuroglu et al., 2003; Ruiz et al., 2005; Zeb & Mehmood, 2004).

The range of fruit‐related characters was as follows: fruit length: 34.99–38.20 mm, fruit width: 31.32–36.88 mm, fruit weight: 27.37–33.99 g, and fruit flesh thickness: 11.16–13.47 mm. The TSS varied between 17.00% and 23.87% (Table 1). Fruit quality‐related characters are fundamental for acceptance of apricot fruits by consumers (Ruiz & Egea, 2008). Thus, fruit quality‐related attributes should be mainly considered for introducing new apricot cultivars. The traits, such as fruit size, texture, firmness, attractiveness, appearance, taste, color, and size, are the main characters related to quality (Abbott, 1999).

Stone length ranged from 28.13 to 30.92 mm, stone width varied from 20.65 to 24.37 mm, stone thickness varied from 10.40 to 11.78 mm, and stone weight varied from 1.48 to 2.71 g. The range of kernel‐related characters was as follows: kernel length: 18.27–20.19 mm, kernel width: 13.62–17.51 mm, kernel thickness: 6.65–7.41 mm, and kernel weight: 0.70–0.81 g. Kernel taste was bitter (17 genotypes), moderate (24), and sweet (7). Recently, sweet kernels of apricots have been used for direct consumption as a snack food like almond. Also, bitter kernels are used in the pharmaceutical and cosmetics industries (Yilmaz et al., 2012). The values of the most important fruit‐related traits for the selected superior late‐blooming genotypes are presented in Table 3.

**TABLE 3:** The most important fruit‐related traits of the superior late‐blooming apricot genotypes selected

Character	Fruit yield	Fruit length (mm)	Fruit width (mm)	Fruit weight (g)	Fruit flesh thickness (mm)	Stone weight (g)	TSS (%)
Shazand−7	High	36.84	36.88	33.99	11.34	1.94	20.04
Khondab−17	High	35.02	31.84	33.53	11.71	1.74	19.76
Marzigharan−8	High	36.62	36.12	33.45	11.16	2.12	21.37
Shazand−3	High	36.51	34.52	33.35	12.61	2.16	21.37
Shazand−15	High	37.07	32.29	33.22	11.61	1.92	20.43
Marzigharan−1	High	35.58	31.32	32.55	11.18	1.99	21.62
Khondab−12	High	35.96	35.49	32.50	13.47	2.13	22.04
Shazand−5	High	35.53	35.31	32.36	13.24	2.27	22.78
Khondab−15	High	35.83	33.09	32.24	13.43	2.13	21.13
Khondab−20	High	35.81	31.59	31.76	12.31	1.71	23.79

Significant correlations were observed between some characters (not shown). Fruit weight was positively and significantly correlated with branch leaf length (*r* = .56), branch leaf width: (*r* = .53), spur leaf length (*r* = .58), spur leaf width (*r* = .51), and fruit length (*r* = .75), and fruit width (*r* = .73) and corresponded with the previous findings (Khadivi‐Khub & Khalili, 2017; Rezaei et al., 2020).

The PCA classified the characters into 18 PCs which justified 81.64% of the total variance (not shown). The PC1 accounted for 6.24% of the total variance and was significantly correlated with tree growth habit, fruit ground color, and fruit flesh color. Three characters, including kernel color, kernel shriveling, and kernel taste, were placed into PC2 and accounted for 6.12% of the total variance. Fruit apex shape and fruit shape were correlated with PC3, which accounted for 5.69% of the total variance. Previously, PCA revealed that fruit‐related characters were important to distinguish the genotypes of apricot (Asma & Ozturk, 2005; Khadivi‐Khub & Khalili, 2017; Mratinic et al., 2011; Rezaei et al., 2020; Ruiz & Egea, 2008).

**FIGURE 1:**
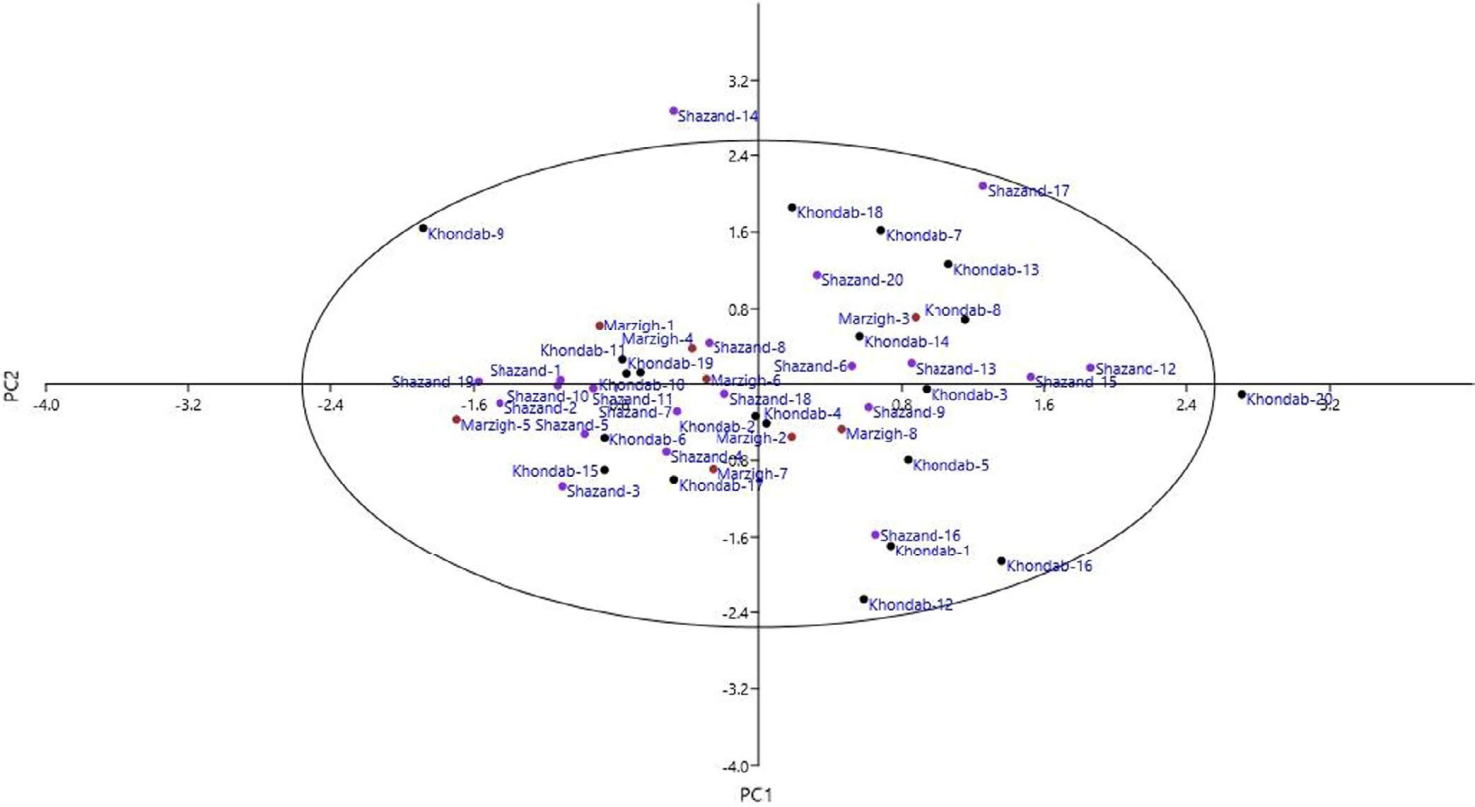
Scatter plot for the studied late‐blooming apricot genotypes based on PC1/PC2. The symbols represent the genotypes of each area in the plot, including Khondab (Kh), Marzigharan (M), and Shazand (Sh)

**FIGURE 2:**
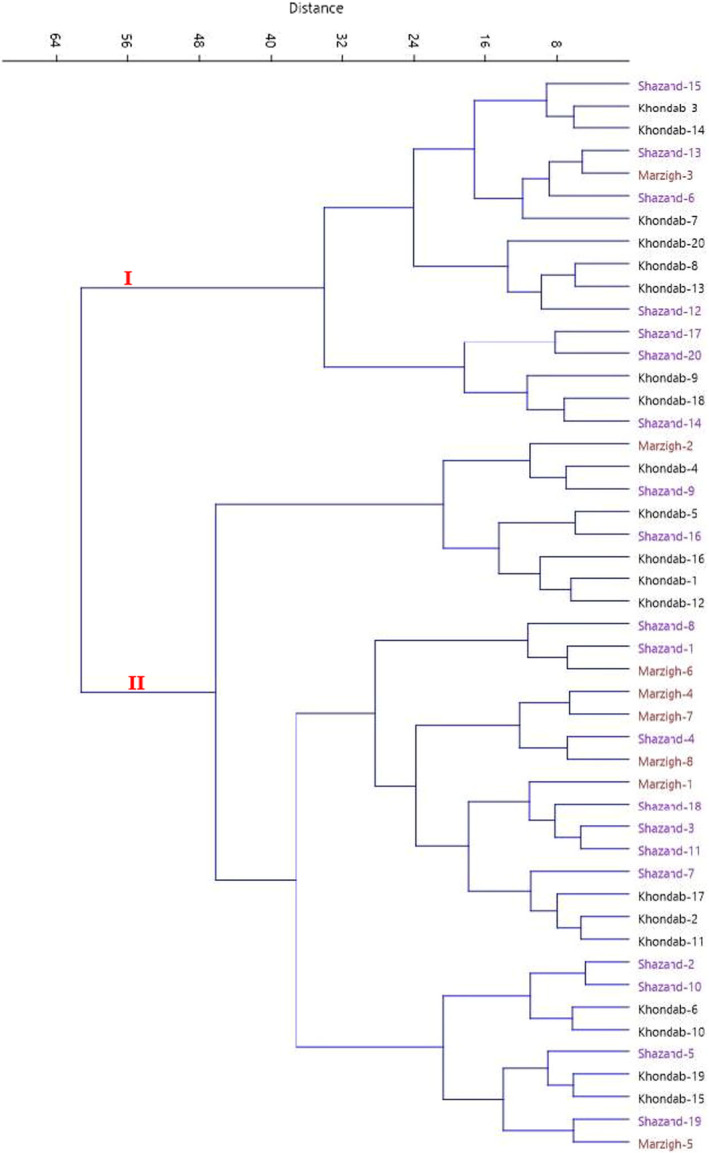
Ward cluster analysis of the studied late‐blooming apricot genotypes based on morphological traits using Euclidean distances

The scatter plot created using PC1/PC2 showed phenotypic variations among the genotypes (Figure 1). The genotypes were distributed into four sides of the plot and showed high differences for most of the characters. Also, the HCA performed with Euclidean distance and Ward's method divided the genotypes into two main clusters based on morphological traits (Figure 2). The first cluster (I) included 16 genotypes, while the second cluster (II) consisted of the rest 32 genotypes. In some cases, the genotypes belonging to an area were clustered into different clusters. Differences in morphological characters under the same environmental and geographical conditions are probably the result of genetic effects (Karadeniz, 2002). The nuclear genome contains the majority of the genes related to different characters and also has high rate of mutation (Provan et al., 2001). Thus, the mutation increases the variation in the population (Khadivi‐Khub et al., 2016).

Late blooming is one of the most important factors in preventing spring frost damages to fruit trees in continental climates. Thus, one of the most important aims in the first phase of apricot breeding programs is to identify and introduce late‐blooming genotypes. Reduction or elimination of damages caused by spring frost is possible by cultivating late‐blooming genotypes or new cultivars having this trait. Besides, because the blooming occurs after the rainy season in such genotypes/cultivars, pollination and effective use of pollinators such as insects significantly increase (Rezaei et al., 2020).

## 4 CONCLUSIONS

In many parts of the world, including Iran, apricot production is limited by late spring frost and thus, late blooming is the most important selection criteria. The knowledge of blooming date and fruit attributes of the apricot genotypes studied here could be useful to choose the appropriate ones to be grown or used as parents in future breeding programs. The promising genotypes were selected through blooming date and then fruit quality‐related characteristics. Thus, after pre‐selections among many genotypes, all the 48 late‐blooming date genotypes selected could be useful as a parent to improve flowering season of cultivars. Furthermore, among them, 10 genotypes, including Shazand‐7, Khondab‐17, Marzigharan‐8, Shazand‐3, Shazand‐15, Marzigharan‐1, Khondab‐12, Shazand‐5, Khondab‐15, and Khondab‐20, were superior in terms of fruit quality‐related characters, such as fruit weight, fruit color, fruit taste, and TSS, and thus they can be singled out for cultivation.

## CONFLICT OF INTEREST

The authors declare that they have no conflict of interest.

## AUTHOR CONTRIBUTIONS

**Zeinab Mashhadi:** Investigation (equal). **Ali Khadivi:** Formal analysis (lead); Methodology (lead); Project administration (lead); Software (lead); Supervision (lead); Validation (lead); Writing – review & editing (lead).

## RESEARCH INVOLVING HUMAN PARTICIPANTS AND/OR ANIMALS

None.

## INFORMED CONSENT

None.

## Data Availability

The data that support the findings of this study are available from the corresponding author upon reasonable request.
